# Remotely supervised spirometry versus laboratory-based spirometry during the COVID-19 pandemic: a retrospective analysis

**DOI:** 10.1186/s12931-023-02586-0

**Published:** 2024-01-18

**Authors:** Łukasz Kołtowski, Mikołaj Basza, Wojciech Bojanowicz, Piotr Dąbrowiecki, Mateusz Soliński, Katarzyna Górska, Piotr Korczyński, Lauren E. Eggert

**Affiliations:** 1 1st Department of Cardiology, Medical University of Warsaw, Warsaw, Poland; 2 Medical University of Silesia, Katowice, Poland; 3 Department of Allergology and Infectious Diseases, Military Institute of Medicine, 04141 Warsaw, Poland; 4 School of Biomedical Engineering & Imaging Sciences, Faculty of Life Sciences & Medicine, King’s College London, Strand, London, WC2R 2LS UK; 5 Department of Internal Medicine, Pulmonary Diseases and Allergy, Medical University of Warsaw, Warsaw, Poland; 6 Division of Pulmonary, Allergy and Critical Care Medicine, Stanford University, Stanford, CA USA

**Keywords:** Spirometry, Pulmonary function tests, Telemedicine, COVID-19

## Abstract

**Background:**

The COVID-19 pandemic has constrained access to spirometry, and the inherent risk of infectious transmission during aerosol-generating procedures has necessitated the rapid development of Remotely Supervised Spirometry (RSS). This innovative approach enables patients to perform spirometry tests at home, using a mobile connected spirometer, all under the real-time supervision of a technician through an online audio or video call.

**Methods:**

In this retrospective study, we examined the quality of RSS in comparison to conventional Laboratory-based Spirometry (LS), using the same device and technician. Our sample included 242 patients, with 129 undergoing RSS and 113 participating in LS. The RSS group comprised 51 females (39.5%) with a median age of 37 years (range: 13–76 years). The LS group included 63 females (55.8%) with a median age of 36 years (range: 12–80 years).

**Results:**

When comparing the RSS group to the LS group, the percentage of accurate Forced Expiratory Volume in one second (FEV1) measurements was 78% (n = 101) vs. 86% (n = 97), p = 0.177; for Forced Vital Capacity (FVC) it was 77% (n = 99) vs. 82% (n = 93), p = 0.365; and for both FEV1 and FVC, it was 75% (n = 97) vs. 81% (n = 92), p = 0.312, respectively.

**Conclusions:**

Our findings demonstrate no significant difference in the quality of spirometry testing between RSS and LS, a result that held true across all age groups, including patients aged over 65 years. The principal advantages of remote spirometry include improved access to pulmonary function tests, reduced infectious risk to curtail disease spread, and enhanced convenience for patients.

## Background

Spirometry remains the most widely employed pulmonary function test (PFT) and the definitive standard for diagnosing and monitoring obstructive lung diseases such as asthma and chronic obstructive pulmonary disease (COPD), the third leading cause of global mortality [1]. The successful execution of this test hinges on substantial patient participation and compliance. A patient's role during the spirometry examination is to attain maximum possible inhalation and exhalation, both in terms of flow and volume. As this necessitates considerable physical exertion, the technician’s role in the spirometry test is vital to effectively support and encourage the patient to achieve maximum effort and maintain correct form.

The COVID-19 pandemic has notably restricted access to PFTs due to the need to prevent the spread of SARS-CoV-2 (severe acute respiratory syndrome coronavirus-2) [2]. The Global Initiative for Chronic Obstructive Lung Disease (GOLD) recommendations have suggested using telemedical tools for conducting spirometry during the COVID-19 pandemic [3]. Concurrently, an array of portable, mobile spirometers has emerged, demonstrating parity with traditional desktop spirometers and offering new possibilities for care providers, while maintaining established test characteristics and quality criteria [4].

In the literature, the terms ‘remote spirometry’ or ‘home spirometry’ are used interchangeably and can be interpreted differently. In this manuscript, ‘home spirometry’ refers to training patients in spirometry techniques during a clinical visit, followed by the patients independently performing these tests at home. The resulting spirometry reports and parameters are typically shared via an online platform or presented to the physician during a subsequent visit. This approach is especially beneficial for patients requiring ongoing monitoring of chronic diseases impacting lung function, such as asthma, COPD, cystic fibrosis, or neuromuscular diseases [5–9]. Given their frequent exposure to these tests, these patient groups often possess significant experience in appropriate spirometry test performance techniques.

However, this manuscript focuses on ‘Remotely Supervised Spirometry’ (RSS), where patients perform the examination under real-time supervision from a technician using telemedicine tools. This method allows the technician to oversee the spirometry examination process similar to an in-person visit, but with the added benefits of telemedicine, including curbing the spread of infectious diseases, removing geographical barriers, enhancing access to professional pulmonary function testing, and reducing costs.

In our study, the RSS protocol involved patients performing a spirometry examination at home, their natural environment, using a mobile-connected spirometer under the real-time supervision of a technician during a video call. Our primary objective was to compare the quality of spirometry examinations conducted at home under a technician's supervision with the results from traditional in-person, laboratory-based spirometry tests performed using the same device and by the same technician.

## Methods

### Study design

This retrospective study compares the quality of spirometry examinations between two groups: (a) the Remotely Supervised Spirometry group (RSS group), consisting of patients who ordered the “Home Spirometry” service in Poland between August 2020 and June 2021 through an online website and performed remotely supervised dynamic spirometry maneuvers according to the protocol detailed below; and (b) the Laboratory-Based Spirometry group, comprising of patients who underwent spirometry in a Warsaw spirometry laboratory in September 2021, as part of a screening program for obstructive pulmonary diseases. In both groups, the spirometry examinations were conducted using the same device and supervised by the same spirometry technician.

### Equipment and operation

The spirometry tests were carried out using a fully-connected portable spirometer (AioCare^®^, HealthUp, Poland), a Class IIa hospital-grade device conforming to the ATS/ERS 2019 quality criteria and the European Union’s General Data Protection Regulations [4]. The device was Bluetooth^®^-connected to the patient’s smartphone (iOS/Android) and operated via a mobile app (AioCare Patient). Patients were asked to create an account and input their biometric (age, sex, ethnicity, weight, height) and basic medical data. The app automatically analyzed the technical quality of all spirometry examinations in real-time according to the ATS/ERS 2019 Standards [10]. All results were automatically uploaded to a secure health cloud platform (AioCare Panel), enabling the technicians to review the flow-volume and volume-time curves in detail between maneuvers.

### Examination protocol

The Laboratory-Based Spirometry was conducted in accordance with the ATS/ERS 2019 Standardization. The technician was required to wear full personal protective equipment (PPE), including a gown, an FFP2 mask, and eye protection. The examination room was thoroughly cleaned and disinfected between patients. Every patient underwent SARS-CoV-2 testing prior to the examination.

The protocol for the remote spirometry test proceeded as follows:

- An antibacterial-filter-equipped mobile spirometer and a nose clip were delivered contact-free to the patient’s home.
- The patient followed the instructions provided in the parcel, downloaded the mobile app (AioCare Patient), created a patient account, and prepared for the spirometry test.
- At the scheduled time, the patient connected with the technician via an online communicator offering video and audio connectivity. No PPE was required for either the patient or the technician.
- After brief instruction, the patient performed a spirometry examination under the technician’s supervision, who had a real-time preview of the patient nad the examination. The results of the examination were instantly visible on the dashboard after the maneuver. The spirometry technician reviewed the results of each maneuver and provided real-time feedback and education to the patient as necessary, focusing on the correct technique for performing the test before subsequent attempts.
- Once three correct maneuvers had been performed and the repeatability criteria had been met, the examination was completed. The patient then returned the spirometer using the provided sealed packaging.

**Fig. 1:**
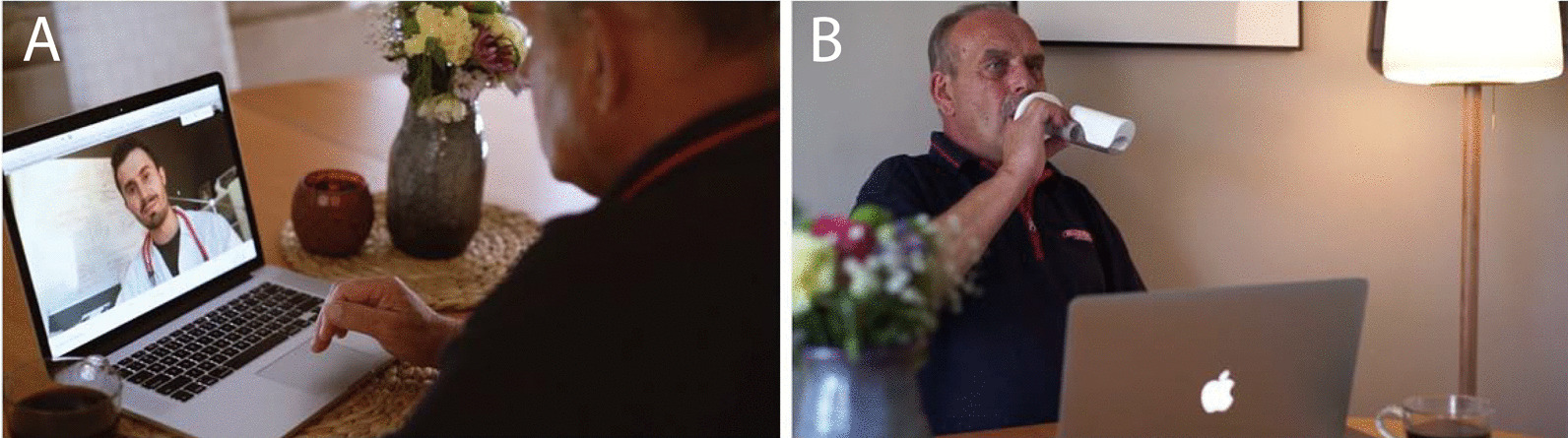
Protocol for Remotely Supervised Spirometry Examination. **A** Technician Instructing and Preparing the Patient Prior to Spirometry Maneuver. **B** Patient Performing Maneuver under Technician’s Supervision via Video Call

In Figure 1 illustrates the video call setup and the configuration of the spirometry test.

### Data analysis

The outcomes of continuous variables were reported as X (Q1–Q3), where X signifies the median and Q1 and Q3 indicate the first and third quartiles, respectively. Differences between the two groups were evaluated using the chi-square test for categorical variables, and the t-test or Mann–Whitney U test was employed for continuous variables, depending on whether the assumption of normality was met. The latter was tested using the Shapiro–Wilk test. A significance level of p < 0.05 was considered statistically significant. The accuracy of the spirometry examination, based on the 2019 ATS/ERS standards [10], was evaluated for FEV1 (forced expiratory volume in the first second) and FVC (forced vital capacity) both independently and collectively (i.e., both FEV1 and FVC needed to meet the pre-established criteria). The reference values for FEV1, FVC and FEV1/FVC were calculated using GLI 2012 recommendations [11]; for PEF we used the ERS reference values [12].

## Results

The RSS group comprised 129 patients (51 females) with a median age of 37 (27–47) years (range: 13–76 years) and a median BMI of 25.1 (22.5–27.6) kg/m^2^. The LS group consisted of 113 patients (63 females) with a median age of 36 (32-41) years (range: 12–80 years) and a BMI of 24.6 (21.7–27.8) kg/m^2^. The study groups were well balanced though the individuals in the RSS group were slightly taller and heavier that resulted in slightly higher FEV1, FVC; with no differences in FEV1/FVC and FEV1/FVC%. The comparative baseline characteristics and average values of spirometry parameters are presented in Table 1.

**Table 1:** Baseline clinical and demographic characteristics

	Remote supervised spirometry (n = 129)	Laboratory-based spirometry (n = 113)	p-value
Age (years)	37 (27–47) (min: 13, max:	36 (32–41) (min: 12, max:	0.608
	76)	80)	
Females, n	51 (39.5%)	63 (55.8%)	0.0167
(%)			
Height (cm)	176 (166–183)	170 (164–178)	0.0042
Weight (kg)	78 (65–90)	71 (62–85)	0.054
BMI (kg/m^2^)	25.1 (22.5–27.6)	24.6 (21.7–27.8)	0.569
FEV1 (L)	3.73 (3.11–4.65)	3.49 (2.95–4.12)	0.093
FVC (L)	4.78 (3.89–5.73)	4.25 (3.67–5.11)	0.017
FEV1/FVC	0.81 (0.76–0.84)	0.81 (0.79–0.85)	0.222
PEF (L/min)	469 (381–589)	422 (360–541)	0.114
FEV1 (%	102 (90–113)	99 (89–106)	0.056
predicted)			
FVC (%	104 (95–115)	99 (91–106)	0.0016
predicted)			
FEV1/FVC	98 (93–102)	99 (96–103)	0.110
(%)			

Data presented as number (percentage); median (Q1-Q3) or as counts

BMI, body mass index; FEV_1_, forced expiratory volume at the first second; FVC, forced vital capacity; SD, standard deviation; PEF, peak expiratory flow

**Fig. 2:**
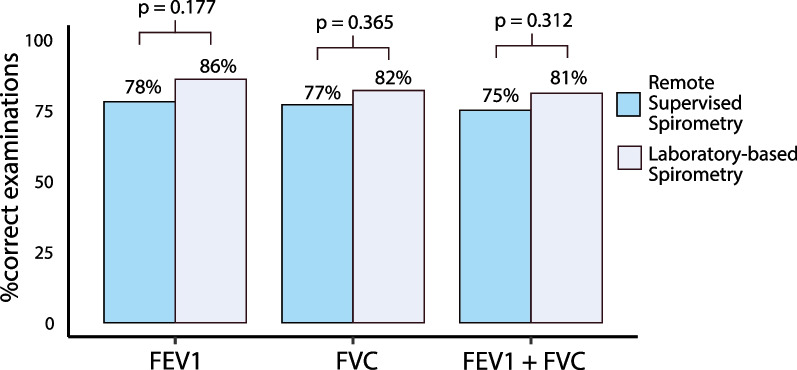
Comparative Analysis of Technical Correctness in Spirometry Examinations

**Fig. 3:**
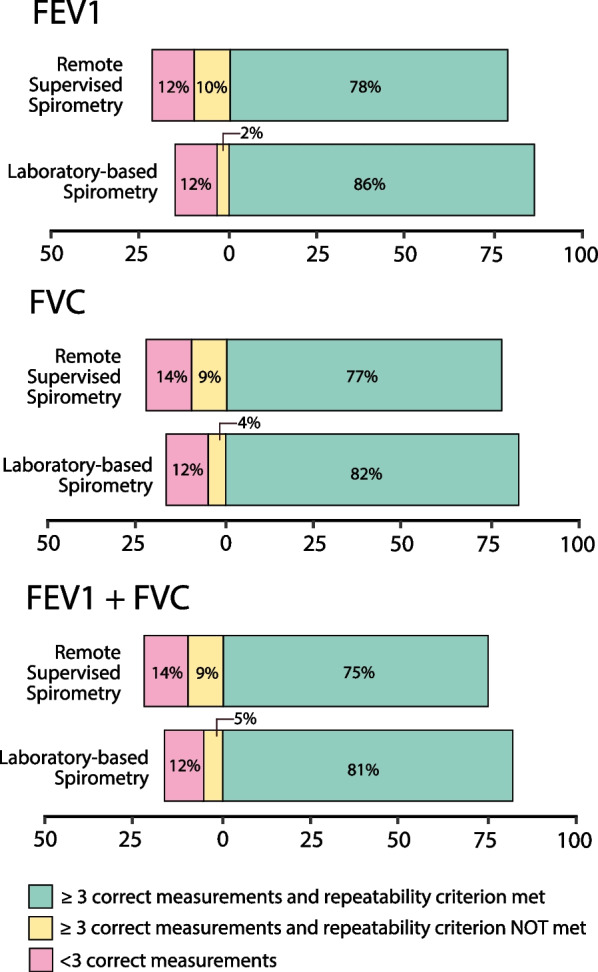
Percentage of examination regarding the correctness and repeatability criteria for FEV1, FVC and FEV1 + FVC

The proportion of spirometry examinations meeting the technical correctness criteria for the FEV1 parameter was 78% (n = 101) in the remote supervised spirometry group vs. 86% (n = 97) in the laboratory spirometry group, p = 0.177; for FVC, 77% (n = 99) vs. 82% (n = 93), p = 0.365, and for both correct FEV1 and FVC (FEV1+FVC), 75% (n = 97) vs. 81% (n = 92), p = 0.312, respectively (Figure 2). No significant differences were observed in the remote spirometry group between patients aged ≤ 40 years and > 40 years (FEV1+FVC criteria: 79% vs 69%, p = 0.235), nor between males and females (73% vs 78%, p = 0.631). Figure 3 displays the number of correctly and incorrectly performed spirometry examinations (lack of repeatability and less than three correct maneuvers) for FEV1, FVC, and FEV1+FVC parameters. We observed higher percentage of the examinations with lack of repeatability in remote supervised spirometry than laboratory spirometry, however, the difference was significant only for FEV1 parameter (FEV1: 10% vs. 2%, p-value = 0.011; FVC: 9% vs. 4%, p-value 0.121; FEV1+FVC: 9% vs. 5%, p-value: 0.228). The percentage of the most frequent errors in single maneuvers for incorrect examinations is presented in Table 2.

**Table 2:** Error frequencies in incorrect maneuvers (FEV1 + FVC)

Error type	Remote supervised spirometry n (%)	Laboratory spirometry n (%)	p-value
TPEF > 300	109/223 (49%)	107/158 (68%)	< 0.001
Ms			
BEV error	24/223 (11%)	35/158 (22%)	0.0039
Plateau error	19/223 (8.5%)	1/158 (0.6%)	0.0015
Cough	4/223 (1.8%)	11/158 (7%)	0.022

TPEF, Time to Peak Expiratory Flow; BEV, Back Extrapolated Volume; FEV1, First Second Expiratory Flow; FVC, Forced Vital Capacity

Significant differences were observed between the laboratory-based and remote supervised spirometry groups regarding individual technical errors. Technical errors were more frequent in the laboratory-based spirometry group for TPEF (time to peak expiratory flow) > 300 ms (68% vs. 49%, p < 0.001), BEV (back extrapolated volume) error (22% vs. 11%, p = 0.0039), and cough (7.0% vs. 1.8%, p = 0.022). In the remotely supervised spirometry group, more patients had difficulty achieving the plateau (0.6% vs 8.5%, p = 0.0015).

## Discussion

Remotely supervised spirometry represents an innovative, telemedical approach to spirometry testing. This method offers enhanced accessibility to spirometry tests, particularly beneficial for immobilised patients or those residing in smaller urban centers without access to specialized pulmonary function labs. It also helps prevent the spread of contagious diseases like COVID-19 and offers convenience for both patients and technicians. Our study demonstrates that remotely supervised spirometry maintains high quality and is comparable to traditional laboratory-based spirometry.

Our findings confirm that a remote monitoring platform can achieve high-quality spirometry at a patient’s home. However, the technician's expertise and patient engagement are still paramount for high-quality spirometry, irrespective of the approach employed. A study by Jankowski et al. reported that only 49% of spirometry examinations performed in primary care offices met the criteria for at least three acceptable maneuvers and repeatability, while 38.2% had at least three acceptable maneuvers but did not meet the repeatability criteria [13]. Compared to these findings, our remote spirometry approach showed a 26% increase in acceptable spirometry examinations. In a Dutch study, Landman et al. demonstrated that 60.3% of examinations were acceptable in primary care diagnostic centers and 31.9% in GP offices [14]. Notably, Landman’s study required only two acceptable maneuvers for acceptance criteria. A similar study by van de Hei et al. reported only 13.4% full adherence to ATS/ERS criteria [15]. Based on these findings, we propose that remotely supervised spirometry could be a viable alternative to office spirometry testing in primary care, offering wider access, higher quality, and lower cost.

Our study identified numerous parallels between laboratory-based spirometry and remotely supervised spirometry performed with online audio/video supervision. In both settings, the patient is instructed and responds via verbal and non-verbal communication, a critical factor for the operator to gauge the patient’s involvement in the examination. Observing the patient through a video call complies with the ATS/ERS 2019 [10] criteria for monitoring the patient’s effort during maneuvers. According to spirometry standards, maximal effort during inhalation may be indicated by signs such as rising eyebrows or head quivering, whereas a comfortable-looking patient during the inhalation maneuver is unlikely to exert sufficient effort. Furthermore, real-time numerical results of consecutive spirometry tests and the patient’s involvement observed during the video interview enable an experienced technician to identify and rectify technical errors that may occur during the test. However, live gestural and verbal communication may be more intuitive and precise than in the case of remote instruction. Factors like data transmission delay during a video call might affect the examination quality and patient-technician communication by impacting the response time to the technician’s commands. This could potentially introduce errors such as TPEF and BEV. Interestingly, in our study, the instances of TPEF (p-value < 0.001) and BEV (p-value = 0.0039) errors were lower in the remote spirometry group, which could be attributed to factors such as patient motivation, safety, and comfort at home (Table 2). ATS/ERS standards highlight laboratory details such as comfort and patient privacy as important factors [10]. In a 2011 study by Masa et al., a comparison was drawn between stationary spirometry and remotely supervised spirometry, which involved a technician in a different room of a pulmonary clinic [16].

The study revealed a comparable correctness rate of 85.5% for stationary spirometry and 79.6% for remote spirometry, with a variation of 5.9% falling within the expected range depending on the technician's experience in traditional spirometry. Importantly, no clinically significant differences were found between the two approaches. However, it is worth noting that the Masa et al. study entailed remote control of a computer with spirometry software by the technician. In contrast, our protocol demanded greater patient engagement, requiring patients to perform spirometry at home while managing pre-test setup, including the installation of the app, account registration, technician connection, and device setup. Furthermore, patients needed to navigate the spirometry software (mobile app) independently during the test. This level of digital competency could significantly influence adherence to ATS/ERS criteria.

Our study found no significant differences between patients over and below 40 years old. However, it should be noted that patients in the remote spirometry group were required to order the service online, suggesting a level of digital fluency. As such, digital exclusion is a key consideration when deploying novel telemedical methods for spirometry examinations. In cases of technical difficulties, the patient could rely on the technician's support and patience and the technical assistance of family members and caregivers at home. This highlights the crucial role of caregivers and family members in the process, especially for elderly patients. Yet, enabling the technician to control the computer or mobile device remotely could potentially make the spirometry examination more accessible and comfortable for the patient. The value of remote spirometry is further underscored in patients with chronic lung diseases requiring consistent, long-term lung function monitoring at home using portable spirometers [5–9]. A study by Kupczyk et al. found that after a three-week follow-up, 96% of patients performed at least one correct examination at home following initial training at the office [8]. Similarly, a single-centre study at the Cystic Fibrosis Clinic at Royal Prince Alfred Hospital, Sydney, Australia, demonstrated that trained adult Cystic Fibrosis patients could perform unsupervised spirometry tests at home with results comparable to those performed under respiratory scientist supervision [6]. Remote spirometry could be an effective tool in the initial training or retraining of inexperienced patients for subsequent home monitoring.

Our study has several limitations. Firstly, being a retrospective, observational study, achieving a perfect balance of demographic and clinical characteristics proved challenging. Although no significant differences were found in the median age across both groups, with similar age ranges also noted, we were constrained by limited clinical baseline characteristics. As such, it can be ruled out that these two populations might have differed in factors not included in our study, such as prior experience with spirometry and levels of education. Therefore a cross-over study in the same patient population would be preferred to minimize the influence of patient characteristics and directly compare both methods. Moreover, randomization to order of tested method would be desirable, to avoid the influence of training just before the next maneuver. Secondly, we did not calculate the sample size for this study, instead opting to include all available patients in our analysis. While this approach lends our study a more real-world data registry feel, it does present certain limitations in terms of statistical power and representativeness. Thirdly, the study does not provide a cost analysis of both diagnostic strategies, which could have offered additional insights for readers. Another limitation of our study was a comparison of a single examination, between two different patient groups. Further studies should evaluate the reproducibility of examination in the same patient group. Future studies could consider further incorporating such an analysis to inform decision-making in this area. Fourthly, our study was single-operator, limiting the generalizability of our findings. Replicating these results in larger, prospective, and preferably randomised studies is necessary to corroborate our findings. Despite these limitations, the evidence provided in this study holds value and should be shared with the broader community and healthcare providers. This study offers initial insights into the potential of remotely supervised spirometry as a feasible and effective method for respiratory assessment, warranting further investigation.

## Conclusions

Remotely supervised spirometry, as a novel telemedical diagnostic modality, enables the acquisition of high-quality spirometry results at home. Our study demonstrates that achieving a high-quality spirometry examination in a remote supervised setting is feasible, with a technical quality level comparable to laboratory-based examinations and likely superior to office spirometry in primary care settings. With its ease of scalability and low cost, remotely supervised spirometry has the potential to address the unmet need for respiratory diagnostics, making it a valuable tool in the evolving landscape of healthcare.

## Data Availability

The datasets used during the current study are available from the corresponding author upon reasonable request.

## Abbreviations

RSS: Remotely supervised spirometry
LS: Laboratory-based Spirometry
FEV1: Forced Expiratory Volume in one second
FVC: Forced vital capacity
PFT: Pulmonary function test
COPD: Chronic obstructive pulmonary disease
SARS-CoV-2: Severe acute respiratory syndrome coronavirus-2
GOLD: The Global Initiative for Chronic Obstructive Lung Disease
PPE: Personal protective equipment
TPEF: Time to peak expiratory flow
BEV: Back extrapolated volume
